# Associations of plasma p‐tau231 with serial position recall performance in free‐of‐dementia individuals

**DOI:** 10.1111/jnp.70012

**Published:** 2025-09-23

**Authors:** Davide Bruno, Chelsea Reichert‐Plaska, Ainara Jauregi‐Zinkunegi, Nicholas J. Ashton, Henrik Zetterberg, Kaj Blennow, Nunzio Pomara

**Affiliations:** ^1^ School of Psychology Liverpool John Moores University Liverpool UK; ^2^ Department of Communication Sciences and Disorders University of Wisconsin‐Madison Madison Wisconsin USA; ^3^ Geriatric Psychiatry Division Nathan Kline Institute Orangeburg New York USA; ^4^ Department of Psychiatry New York University Grossman School of Medicine New York New York USA; ^5^ Department of Psychiatry and Neurochemistry Institute of Neuroscience and Physiology, the Sahlgrenska Academy at the University of Gothenburg Mölndal Sweden; ^6^ Banner Health Phoenix Arizona USA; ^7^ Clinical Neurochemistry Laboratory Sahlgrenska University Hospital Mölndal Sweden; ^8^ Department of Neurodegenerative Disease UCL Institute of Neurology London UK; ^9^ UK Dementia Research Institute at UCL London UK; ^10^ Hong Kong Center for Neurodegenerative Diseases Hong Kong China; ^11^ Wisconsin Alzheimer's Disease Research Center University of Wisconsin School of Medicine and Public Health, University of Wisconsin‐Madison Madison Wisconsin USA; ^12^ Paris Brain Institute, ICM, Pitié‐Salpêtrière Hospital Sorbonne University Paris France; ^13^ Division of Life Sciences and Medicine, and Department of Neurology, Neurodegenerative Disorder Research Center, Institute on Aging and Brain Disorders University of Science and Technology of China and First Affiliated Hospital of USTC Hefei P.R. China; ^14^ Department of Pathology New York University Grossman School of Medicine New York New York USA

**Keywords:** Alzheimer's disease, memory assessment, plasma biomarkers

## Abstract

Cognitive assessment and analysis of plasma biomarkers are lower‐cost options for the early assessment of Alzheimer's disease (AD). In this study, we examined whether serial position markers in the Rey's AVLT were sensitive to plasma AD biomarkers in cognitively unimpaired older individuals. Participants (*n* = 327; mean age = 70.4, SD = 10.4) were free of dementia (MMSE = 24+) at baseline and recruited as part of the Memory Evaluation Research Initiative (MERI; Nathan Kline Institute, NY, USA). Data included plasma p‐tau231, Aβ40 and Aβ42, AVLT scores and demographics. Bayesian linear and logistic regression analyses were carried out with plasma biomarkers as outcomes (including the Aβ42/40 ratio); memory scores, including traditional metrics and serial position scores, were predictors; and age, years of education, *APOE* ε4‐status and reported gender were control variables. Results indicated that plasma p‐tau231 was associated primarily with delayed primacy recall (first four words): the more primacy words were recalled, the lower the plasma p‐tau231 levels were. This study confirms that serial position analysis of word‐list recall data, and particularly delayed primacy, is a valuable tool for the identification of in vivo AD‐related pathology in cognitively unimpaired individuals.

## INTRODUCTION

Early detection of Alzheimer's disease (AD) is one of the critical components of the global response to the growing dementia crisis. Accurate, affordable and accessible detection tools are needed to address the increasing prevalence of dementia in low‐ and middle‐income countries. Plasma biomarkers are developing into a very attractive tool for early screening and diagnostics as they are lower‐cost options for early diagnostics compared to positron emission tomography (PET) imaging and less invasive alternatives than lumbar puncture. However, plasma testing may still be difficult to access within rural communities and remote locations (Anticona Huaynate et al., 2015), which presents with a series of practical challenges when implementing in clinical practice (Della Monica et al., 2024; Schöll et al., 2024).

A second alternative to PET imaging and lumbar puncture is cognitive assessment. Cognitive assessment provides direct information on an individual's cognitive state, but only indirect information about their possible underlying neuropathology. Moreover, cognitive assessment is relatively inexpensive and, in some cases, shows good concordance with biomarker results (Bruno, Jauregi Zinkunegi, Kollmorgen, et al., 2023; Bruno, Jauregi Zinkunegi, Pomara, et al., 2023). One way to improve cognitive assessment sensitivity to underlying AD and related dementias neuropathology is to adopt item‐level analysis of cognitive responses (Bruno et al., 2024). Item‐level analysis requires the assessment of item‐by‐item responses of participants and aims at identifying the underlying neurocognitive processes leading to the cognitive performance (Mueller et al., 2022), in tune with the process approach (Libon et al., 2013; Milberg et al., 2009).

Analysis of serial position performance, where memory is better for stimuli learned at the beginning (primacy) and/or at the end (recency) of a list, is one type of process scoring applied to word‐list recall that improves detection of AD pathology over traditional metrics (Bruno, Jauregi Zinkunegi, Kollmorgen, et al., 2023). For example, delayed primacy from a word‐list test has been found to be associated with global AD pathology and neuritic plaques, which are the consequence of amyloid‐β protein aggregation (Bruno et al., 2024), while loss of recency in both word lists and stories has been linked to higher levels of CSF tau levels (Bruno, Jauregi Zinkunegi, Pomara, et al., 2023; Bruno, Zinkunegi, et al., 2023).

In this study, we examined whether serial position markers in the Rey's auditory verbal learning test (AVLT; Rey, 1983) were sensitive to cross‐sectional plasma AD biomarker levels in a cohort of community‐dwelling free‐of‐dementia older adults: the Memory Evaluation Research Initiative (MERI) from the Nathan Kline Institute (NY, USA). We focused specifically on the relationship between serial position performance and plasma phosphorylated (p)‐tau231. Evidence shows that p‐tau231 may be the first isoform to respond to brain tau pathology (Antonioni et al., 2023), but few studies have examined how this marker is related to cognitive performance in individuals who are free of dementia (Martínez‐Dubarbie et al., 2024). Secondary analyses also examined the relationship between serial position scores in AVLT and plasma levels of amyloid β (Aβ) 40 and 42 (including the 42/40 ratio).

## METHODS

### Sample

A total of 725 MERI participants completed an evaluation, including AVLT and a blood draw for plasma determination. In a first set of analyses, 327 participants were included after applying the following criteria: participants had to be free of dementia, as determined by a Mini‐mental State Exam (MMSE; Folstein et al., 1983) score of 24 or higher (Creavin et al., 2016; Rohden et al., 2025); participants' data had to include plasma p‐tau231, Aβ40 and Aβ42, and AVLT performance at the same visit, plus demographic data. Average age was 70.4 years (SD = 10.4) and 188 individuals were female (57.5%; see Table 1 for more information).

**TABLE 1:** Mean and standard deviation (SD) of p‐tau231, Aβ40, Aβ42 (in pg/mL) and Aβ42/40 ratio; demographics variables; mini‐mental state exam (MMSE) score; AVLT scores, including serial position and the learning ratio.

	MMSE 24+ (mean, SD)	MMSE 26+ (mean, SD)
p‐tau231	8.69, 5.13	8.43, 5.14
Aβ40	97.31, 33.41	97.15, 32.41^a^
Aβ42	5.87, 2.10	5.91, 2.09^a^
Aβ42/40 ratio	0.06, 0.01	0.06, 0.01^a^
Age	70.43, 10.36	69.64, 10.33
Education	15.60, 2.83	15.81, 2.73
MMSE	28.37, 1.70	28.73, 1.26
AVLT total	40.77, 13.24	42.56, 12.46
AVLT delayed	6.80, 4.47	7.35, 4.31
Trial1 primacy	1.79, 1.18	2.04, 3.12
Trial1 middle	1.83, 5.13	1.94, 5.24
Trial1 recency	1.99, 1.13	2.02, 1.11
Delayed primacy	2.09, 1.45	2.24, 1.39
Delayed middle	3.16, 2.29	3.44, 2.24
Delayed recency	1.55, 1.31	1.67, 1.31
Learning ratio	0.50, 0.28	0.53, 0.28

	Frequency	Frequency
Gender (# of females)	188 (57.5%)	178 (57.2%)
*APOE* ε4‐status	106 (32.4%)	96 (30.9%)

*Note*: On the left are data for the MMSE 24+ sample (*n* = 327), and on the right are data for the MMSE 26+ sample (*n* = 311). Also reported are frequency and % of reported female gender and *APOE* ε4‐status.

^a^ Data from 299 participants due to missing scores.

Second, to provide a sensitivity check, we replicated the main analysis, including only participants whose MMSE score was 26+, while all other criteria remained the same. This sample was 311 participants; the average age was 69.6 (SD = 10.3), and females were 178 (57.2%).

The complete MERI procedures have been discussed previously (Reichert et al., 2015), but briefly: MERI is a community‐based programme offering residents of Rockland County, NY, USA, free clinical and neuropsychological assessment and evaluation. Most volunteers take part in MERI due to age‐related concerns with their memory or because of a family history of dementia. The MERI programme was reviewed and approved by the Nathan S. Kline Institute/Rockland Psychiatry Center Institutional Review Board. All participants provided informed consent.

### Cognitive assessment

The AVLT comprises 15 semantically unrelated words read and tested over five learning trials; after about 20 minutes, a delayed recall test is also performed. Traditional memory metrics in use were total recall (over the five learning trials) and delayed recall. Serial position metrics, as per previous papers (e.g. Bruno et al., 2013; Talamonti et al., 2020), were the number of words recalled at the primacy (first four), middle (middle seven) and recency (last four) positions in the first (immediate recall) and delayed trials. We also wanted to compare serial position metrics with the learning ratio, a score indexing forgetting (Boscarino et al., 2024). The learning ratio is calculated using this formula, as applied to AVLT: (final learning trial (5th) recall – first learning trial recall)/(15 – first learning trial recall); to note, 15 is the total number of items to recall in each trial.

### Blood biomarkers

Plasma concentrations of Aβ40 and Aβ42 were measured using the Neuro‐4‐Plex‐E Single molecule array (Simoa) assay (Quanterix, Billerica, MA). Plasma p‐tau231 concentration was measured by an in‐house Simoa assay (Ashton et al., 2021).

### Analysis plan

Bayesian linear regression analyses were carried out with p‐tau231, Aβ40, Aβ42 and the Aβ42/40 ratio, as outcomes, in separate analyses; memory scores, including traditional metrics and serial position scores, were used as predictors; we also compared serial position metrics with the learning ratio. Age, years of education, *APOE* ε4‐status, MMSE score and reported gender were used as control variables, forming the *null* model. Credible intervals (CIs) were set to 95%. The prior was set to the Jeffreys‐Zellner‐Siow prior, which assigns a normal distribution to each regression coefficient and is recommended for Bayesian regression analyses (Heo & Van de Schoot, 2020). The model prior was set to Uniform, which assigns equal prior probabilities to all models under consideration. One thousand Markov chain Monte‐Carlo simulations were conducted to determine parameters and compensate for possible violations of normality. Following that, a post‐hoc Bayesian logistic regression analysis was conducted comparing people who exceeded the clinical p‐tau231 cut‐off of 17.7 with those below to determine utility for diagnostic purposes. The main predictor from the initial analysis was applied alongside the same covariates. Analyses were conducted using JASP (0.18.3; https://jasp‐stats.org/).

## RESULTS

### MMSE 24+

**FIGURE 1:**
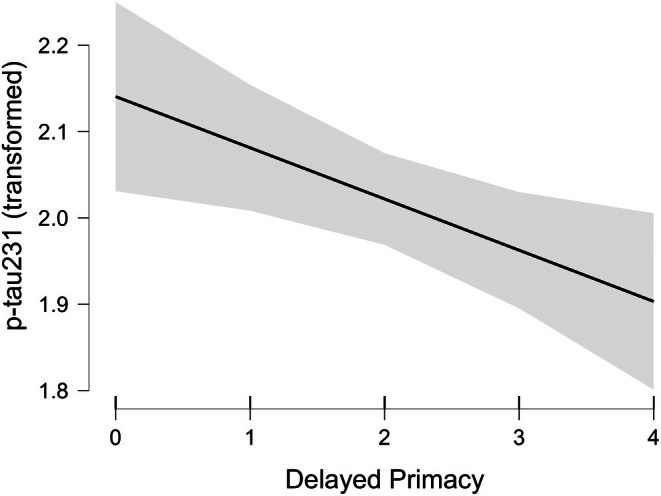
Relationship between transformed plasma p‐tau 231 levels (natural log) and delayed primacy recall (with 95% confidence intervals).

Visual inspection of q‐q plots suggested that the plasma p‐tau231 outcome should be adjusted. A natural‐log transformation yielded q‐q plots indicating a normal distribution. The linear regression analysis on the transformed data suggested the best fitting model had only delayed primacy recall (moderate evidence: BF_10_ = 4.6, BF_inclusion_ = 1.2; see Figure 1). The second‐best model (BF_10_ = 3.8) included delayed recall. Following this analysis, the test coefficient for delayed primacy was calculated over the untransformed plasma p‐tau231, after removal of the other predictors. The fewer delayed primacy words that were recalled cross‐sectionally, the higher the plasma p‐tau231 levels were (mean coefficient = −0.54, SD = 0.27; one fewer delayed primacy word recalled corresponded to approximately 6% higher plasma p‐tau231; CIs: −1 to 0, i.e. 12% to 0%).

The Bayesian logistic regression (22 of 327 individuals exceeded the p‐tau231 cutoff; 7%; see Table 2 for a comparison across sub‐groups) confirmed these findings: lower levels of delayed primacy were associated with a higher risk of positive classification (moderate evidence; BF_10_ = 4.8). Diagnostic estimates were also derived. In general, adding delayed primacy to the model with the covariates increased the area under the curve from 0.73 to 0.77. When setting a low delayed primacy cut‐off of 0.5, essentially comparing those who scored 0 (i.e. complete failure to recall any item from the primacy region) to those who scored 1–4 (any item recalled), we obtained 81% specificity (i.e. correct rejection, or true negative, rate), 50% sensitivity (i.e. hit, or true positive, rate), 96% negative predictive value (NPV; i.e. the probability that a negative response is a correct rejection rather than a miss, i.e. a false negative) and 16% positive predictive value (PPV; i.e. the probability that a positive response is a hit rather than a false alarm, i.e. a false positive). In contrast, when setting a higher cut‐off of 3.5, comparing perfect performers vs. everyone else, we obtained 47% specificity, 82% sensitivity, 97% NPV and 10% PPV.

**TABLE 2:** Mean and standard deviation (SD) of p‐tau231, Aβ40, Aβ42 (in pg/mL) and Aβ42/40 ratio; demographic variables; mini‐mental state exam (MMSE) score; AVLT scores, including serial position and the learning ratio.

	Negative	Positive
p‐tau231	7.69, 3.36	22.55, 5.42
Aβ40	96.25, 32.20	112.08, 45.51
Aβ42	5.84, 2.10	6.28, 2.12
Aβ42/40 ratio	0.06, 0.01	0.06, 0.01
Age	69.92, 10.41	77.41, 6.66
Education	15.58, 2.83	15.86, 2.90
MMSE	28.41, 1.70	27.77, 1.63
AVLT total	41.46, 13.06	31.18, 12.09
AVLT delayed	7.00, 4.46	4.00, 3.74
Trial1 primacy	1.84, 1.19	1.14, 0.89
Trial1 middle	1.89, 5.29	1.00, 1.38
Trial1 recency	1.99, 1.12	2.05, 1.29
Delayed primacy	2.17, 1.42	1.05, 1.36
Delayed middle	3.25, 2.28	1.96, 2.04
Delayed recency	1.59, 1.32	1.00, 1.07
Learning ratio	0.51, 0.28	0.32, 0.19

	Frequency	Frequency
Gender (# of females)	175 (57.4%)	13 (59.1%)
*APOE* ε4‐status	99 (32.5%)	7 (31.8%)

*Note*: On the left are data for p‐tau231 *negative* participants (*n* = 305), and on the right are data for p‐tau231 *positive* participants (*n* = 22). Also reported are frequency and % of reported female gender and *APOE* ε4‐status.

Plasma Aβ40 and Aβ42 levels were adjusted by the square root to improve q‐q plots, as the natural log transformation did not fix the issue; the ratio scores were left untransformed as their q‐q plots were fine. However, no model was better than the null model for any of these outcomes, indicating that no AVLT metric was a good predictor.

### MMSE 26+ sensitivity check

**FIGURE 2:**
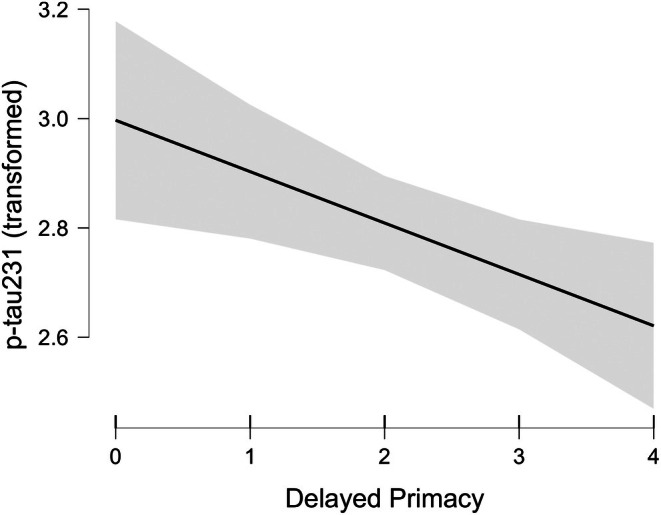
Relationship between transformed plasma p‐tau 231 levels (square root) and delayed primacy recall (with 95% confidence intervals).

The natural log transformation with this sample did not produce normal q‐q plots, so we applied a square root transformation to the p‐tau231 values, which yielded a better plot. The linear regression analysis on the square‐root‐transformed data indicated that the best fitting model had delayed primacy recall alone (moderate evidence: BF_10_ = 5.304, BF_inclusion_ = 1.0; see Figure 2). Following this analysis, the test coefficient for delayed primacy was calculated over the untransformed plasma p‐tau231, after removal of the other predictors. The fewer delayed primacy words were recalled cross‐sectionally, the higher the plasma p‐tau231 levels were (mean coefficient = −0.30, SD = 0.35; one fewer delayed primacy word recalled corresponded to approximately 4% higher plasma p‐tau231; CIs: −1.0 to 0, i.e. 12% to 0%).

## DISCUSSION

With this cohort study of free‐of‐dementia elderly volunteers, we set out to test whether AVLT‐derived serial position metrics were sensitive to plasma p‐tau231 levels cross‐sectionally. Our results show that delayed primacy recall, more than any other portion of the serial curve or than traditional AVLT metrics, was associated with plasma p‐tau231 levels in this population. As plasma p‐tau231 levels have been shown to be sensitive to the earliest stages of AD pathology (Antonioni et al., 2023; Varma et al., 2024), increasing at small amounts of brain amyloidosis as assessed by amyloid PET (Milà‐Alomà et al., 2022), it is arguable, consistent with previous publications (Bruno et al., 2024), that delayed primacy recall in word‐list learning tests may be a useful tool to identify individuals at risk of AD pathology early and *prior* to further testing.

This study was carried out on up‐to‐date data from MERI and using the AVLT as the word‐learning task. As such, the data were derived from different participants compared to recent publications using serial position analyses from our team (cf. Rush Alzheimer's Disease Center databases; Bruno et al., 2024; Wisconsin Registry for Alzheimer's Prevention; Bruno, Jauregi Zinkunegi, Kollmorgen, et al., 2023; Bruno, Jauregi Zinkunegi, Pomara, et al., 2023); and using a different test (cf. CERAD; Bruno et al., 2024). In addition, we examined a different biomarker outcome (i.e. plasma p‐tau231). We believe that it is important for research investigating cognitive metrics to attempt replication with and generalisation to different cohorts, tests, and outcomes.

Diagnostics derived from the analyses suggest that delayed primacy performance in the AVLT provides consistently high NPVs but also consistently low PPVs. However, the low prevalence of plasma p‐tau231 positivity, likely because participants were free of dementia, makes these diagnostic scores less reliable, especially PPV especially (Ranganathan & Aggarwal, 2018). Prevalence‐free measures of specificity and sensitivity were affected by the cut‐off applied. Setting a low cut‐off of 0.5 favoured specificity (81%), whereas a higher cut‐off of 3.5 favoured sensitivity (82%). Depending on needs, that is wanting to reduce false positives or increase true positives, respectively, different solutions may therefore be therefore preferable.

Limitations should be noted. First, due to the absence of other p‐tau plasma biomarkers, in the available data, we were unable to compare the association of delayed primacy and p‐tau231 to possible associations with other markers, such as p‐tau181 and p‐tau217. Future studies should assess whether AVLT‐based serial position scores, such as delayed primacy, provide better correlations than traditional AVLT metrics to a range of plasma biomarkers in similar cohorts. Second, it should be noted that the vast majority (84%) of the sample self‐reported as White‐European. The lack of diversity, which is unfortunately common in much of AD and related dementia research (Heng & Rittman, 2023; Vyas et al., 2018), makes it impossible in our study to evaluate potential differences across diverse groups of participants. Future research and recruitment within longitudinal cohorts should attempt to address this significant limitation. Third, we defined the lack of overall cognitive impairment with MMSE alone due to a scarcity of other data available for better characterization of the study cohort – ideally, more indices of general cognitive ability are available to obtain a better profile of the participant. Finally, the AUC we reported only reached 0.77, suggesting a potentially sub‐optimal diagnostic measure. However, as p‐tau231 may increase earlier in AD development compared to other blood biomarkers, our findings may suggest that AVLT delayed primacy could be a useful metric for early risk identification, rather than a diagnostic tool per se.

In summary, this study confirms that delayed primacy performance in word‐list recall may be affected in the early stages of AD‐related pathology; it may also be useful in settings where no biomarker testing is available or synergistically with plasma biomarkers to detect clinically relevant brain Aβ and tau pathology.

## AUTHOR CONTRIBUTIONS

**Davide Bruno:** Conceptualization; investigation; writing – original draft; methodology. **Chelsea Reichert‐Plaska:** Conceptualization; writing – review and editing; methodology; data curation; supervision. **Ainara Jauregi‐Zinkunegi:** Writing – review and editing; methodology. **Nicholas J. Ashton:** Methodology. **Henrik Zetterberg:** Methodology; conceptualization; funding acquisition. **Kaj Blennow:** Methodology; funding acquisition. **Nunzio Pomara:** Writing – review and editing; conceptualization; funding acquisition.

## FUNDING INFORMATION

MERI is partially funded by Rockland County, New York, Office of Mental Health, awarded to NP. HZ is a Wallenberg Scholar and a Distinguished Professor at the Swedish Research Council supported by grants from the Swedish Research Council (#2023‐00356; #2022‐01018 and #2019‐02397), the European Union's Horizon Europe research and innovation programme under grant agreement No 101053962, Swedish State Support for Clinical Research (#ALFGBG‐71320), the Alzheimer Drug Discovery Foundation (ADDF), USA (#201809‐2016862), the AD Strategic Fund and the Alzheimer's Association (#ADSF‐21‐831376‐C, #ADSF‐21‐831381‐C, #ADSF‐21‐831377‐C, and #ADSF‐24‐1284328‐C), the European Partnership on Metrology, co‐financed from the European Union's Horizon Europe Research and Innovation Programme and by the Participating States (NEuroBioStand, #22HLT07), the Bluefield Project, Cure Alzheimer's Fund, the Olav Thon Foundation, the Erling‐Persson Family Foundation, Familjen Rönströms Stiftelse, Stiftelsen för Gamla Tjänarinnor, Hjärnfonden, Sweden (#FO2022‐0270), the European Union's Horizon 2020 research and innovation programme under the Marie Skłodowska‐Curie grant agreement No 860197 (MIRIADE), the European Union Joint Programme – Neurodegenerative Disease Research (JPND2021‐00694), the National Institute for Health and Care Research University College London Hospitals Biomedical Research Centre and the UK Dementia Research Institute at UCL (UKDRI‐1003). KB is supported by the Swedish Research Council (#2017‐00915 and #2022‐00732), the Swedish Alzheimer Foundation (#AF‐930351, #AF‐939721, #AF‐968270 and #AF‐994551), Hjärnfonden, Sweden (#FO2017‐0243 and #ALZ2022‐0006), the Swedish state under the agreement between the Swedish government and the County Councils, the ALF‐agreement (#ALFGBG‐715986 and #ALFGBG‐965240), the European Union Joint Program for Neurodegenerative Disorders (JPND2019‐466‐236), the Alzheimer's Association 2021 Zenith Award (ZEN‐21‐848495), the Alzheimer's Association 2022‐2025 Grant (SG‐23‐1038904 QC), La Fondation Recherche Alzheimer (FRA), Paris, France, the Kirsten and Freddy Johansen Foundation, Copenhagen, Denmark and Familjen Rönströms Stiftelse, Stockholm, Sweden.

## CONFLICT OF INTEREST STATEMENT

DB, CRP, AJZ, NA and NP have nothing to declare. HZ has served at scientific advisory boards and/or as a consultant for Abbvie, Acumen, Alector, Alzinova, ALZPath, Amylyx, Annexon, Apellis, Artery Therapeutics, AZTherapies, Cognito Therapeutics, CogRx, Denali, Eisai, LabCorp, Merry Life, Nervgen, Novo Nordisk, Optoceutics, Passage Bio, Pinteon Therapeutics, Prothena, Red Abbey Labs, reMYND, Roche, Samumed, Siemens Healthineers, Triplet Therapeutics and Wave; has given lectures in symposia sponsored by Alzecure, Biogen, Cellectricon, Fujirebio, Lilly, Novo Nordisk, and Roche; and is a co‐founder of Brain Biomarker Solutions in Gothenburg AB (BBS), which is a part of the GU Ventures Incubator Programme (outside submitted work). KB has served as a consultant and at advisory boards for Abbvie, AC Immune, ALZPath, AriBio, BioArctic, Biogen, Eisai, Lilly, Moleac Pte. Ltd., Neurimmune, Novartis, Ono Pharma, Prothena, Roche Diagnostics and Siemens Healthineers; has served at data monitoring committees for Julius Clinical and Novartis; has given lectures, produced educational materials and participated in educational programmes for AC Immune, Biogen, Celdara Medical, Eisai and Roche Diagnostics; and is a co‐founder of Brain Biomarker Solutions in Gothenburg AB (BBS), which is a part of the GU Ventures Incubator Programme, outside the work presented in this paper.

## Data Availability

The data that support the findings of this study are available on request from the corresponding author. The data are not publicly available due to privacy or ethical restrictions.
